# Sharing a flexible urethral sparing SBRT prostate RapidPlan model and quantifying plan quality via dosimetric scorecard with clinical implementation

**DOI:** 10.1186/s13014-025-02761-1

**Published:** 2025-12-17

**Authors:** Jonathan Sackett, Anthony Magliari, Ryan Clark, Lesley Rosa, Kenny Guida, Riqiang Gao, Simon Arberet, Ali Kamen

**Affiliations:** 1 Varian Medical Affairs, Siemens Healthineers, Palo Alto, CA USA; 2 Digital Technology and Innovation, Siemens Healthineers, Princeton, NJ USA; 3 Department of Radiation Oncology, University of Cincinnatti Medical Center, Cincinnati, OH USA; 4 Department of Radiation Oncology, University of Kansas Cancer Center, Kansas City, KS USA; 5 Present Address: Department of Radiation Oncology, University of Arizona Cancer Center, Tucson, AZ USA

**Keywords:** Knowledge-based planning, Prostate SBRT, Dosimetric scorecard, RapidPlan, Treatment planning

## Abstract

**Purpose:**

To develop and validate a Knowledge-Based Planning (KBP) model for prostate stereotactic body radiation therapy (SBRT) using a comprehensive dosimetric scorecard, aiming to improve plan quality and consistency while meeting the stringent criteria of the Adaptive Radiation Therapy Individualized Approach - Prostate (ARTIA) trial.

**Methods:**

A KBP model (ProstateSBRT-ARTIA-SIB40-36 Gy v1.1) was developed using RapidPlan v15.6 with 41 carefully selected prostate SBRT cases. A 35-metric dosimetric scorecard was created to guide the model development and evaluation process. The model was trained iteratively, with the scorecard used to tune optimization objectives and priorities. 10 independent cases were used for validation, testing both VMAT and IMRT beam arrangements.

**Results:**

The KBP model demonstrated significant improvement in plan quality compared to original clinical plans, with the average scorecard score increasing from 166.2/229 to 197.4/229 points. Validation cases showed consistent high-quality plan generation for both VMAT and IMRT techniques. The model exhibited flexibility in adapting to various dose levels and clinical scenarios, including the ability to scale urethral dose constraints.

**Conclusion:**

The dosimetric scorecard-guided KBP model for prostate SBRT demonstrates the potential to generate high-quality treatment plans efficiently and consistently. This approach offers a powerful method for translating complex clinical intent into actionable planning objectives, potentially improving treatment quality and reducing inter-planner variability. Future work will focus on expanding the model’s capabilities, including boosting gross disease volumes and exploring direct optimization based on comprehensive dosimetric scorecards.

## Introduction

Stereotactic body radiation therapy (SBRT) for prostate cancer has gained popularity due to its ability to deliver high radiation doses in fewer fractions, potentially improving treatment outcomes while maintaining acceptable toxicity profiles. However, creating high-quality SBRT plans requires a precise balance of target coverage while sparing organs at risk (OARs) such as the rectum, bladder, and urethra [1]. This challenge necessitates efficient and reproducible treatment planning methods that ensure consistent adherence to clinical trial constraints.

Knowledge-based planning (KBP) has emerged as a powerful tool to improve plan quality, standardize planning processes, and reduce inter-planner variability [2, 3]. Varian RapidPlan can generate high-quality plans by predicting the Dose Volume Histogram (DVH) and relative optimization objectives after being trained from existing high-quality treatment plans. [4]. Site-specific KBP models have been shown to outperform generalized models in terms of plan quality and consistency [5–7].

This study focuses on developing and validating a prostate SBRT KBP model specifically designed to meet the requirements of the Adaptive Radiation Therapy Individualized Approach - Prostate (ARTIA) trial. Max point dose to the urethra has been shown to correlate with acute urinary toxicity [8, 9], and thus ARTIA enforces a urethral dose constrained to $0.1cc < 35Gy$. The KBP model was trained using an iterative refinement process, where optimization objectives were adjusted based on dosimetric scorecard-derived feedback to progressively improve plan quality. A comprehensive 35-metric dosimetric scorecard was incorporated to objectively assess and quantify plan quality, ensuring the model’s flexibility and adaptability to different clinical scenarios [10].

Several studies have demonstrated that an iterative model creation process significantly improves KBP model accuracy, with retrained RapidPlan models producing superior dosimetric outcomes compared to their initial versions [2, 7]. Furthermore, previous work has explored the role of dosimetric scorecards in model optimization and evaluation, demonstrating their ability to guide objective evaluation of treatment plan quality [10–13]. These studies emphasize the importance of scorecards in defining clinical priorities, enabling structured decision-making in KBP model development, and ensuring consistent plan assessment.

The primary objectives of this study are to develop a KBP model for prostate SBRT using a dosimetric scorecard-guided approach, quantify improvements in plan quality compared to clinical plans, and validate model performance on independent test cases. The model was used in plan generation for an automated treatment planning pipeline, demonstrating its applicability in producing consistent, high-quality SBRT plans using the same process as Gao et al. [14]. Additionally, this study evaluates the model’s flexibility in adapting to differing planning intents, particularly those outlined in the ARTIA trial, while ensuring consistency and reproducibility in plan optimization.

## Methods and materials

### Model development

ProstateSBRT-ARTIA-SIB40-36 Gy v1.1 KBP model [15] was developed using RapidPlan v15.6 (Varian Medical Systems, Palo Alto, CA) for maximum compatibility. This model was designed per ARTIA trial, which includes aggressive margins and urethral sparing dose constraints of D0.1cc < 35 Gy. The training dataset consisted of 41 prostate SBRT 6-arc Halcyon cases, carefully selected to represent a range of anatomical variations, prostate sizes, and treatment scenarios. Half the training set cases used rectal spacers, from multiple ARTIA trial enrolled institutions, while the no-spacer patients were not from ARTIA trial institutions.

### Dosimetric scorecard design

A comprehensive 35-metric dosimetric scorecard [15, 16]. was created with input from multiple physicians, including ARTIA trial authors. This scorecard incorporated DVH-based Dose at Volume and Volume at Dose metrics, with relative points assigned on piecewise linear scoring functions for each metric. When summed, these metrics provide a single quantitative score out of 229 points, representing dosimetric plan quality, available in-full in the supplementary material shared online.

**Fig. 1:**
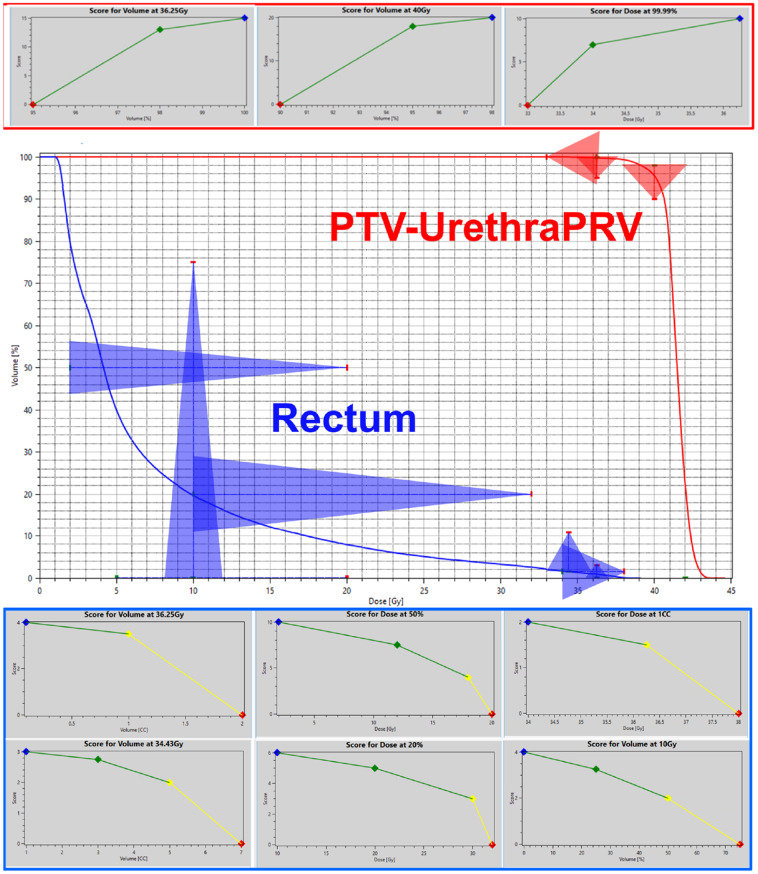
Selected metrics from the dosimetric scorecard used to evaluate ProstateSBRT-ARTIA-SIB40-36 Gy v1.1 KBP model performance. Piecewise linear functions for PTV-UrethraPRV (top) and rectum (bottom). DVH curves on representative case displayed with relative score value triangles overlay (middle). The red point of the triangle corresponds to the zero point on the piecewise linear function

Using dosimetric scorecards for guiding KBP model development has been successfully implemented in various disease sites. [4, 10] The scorecard specifically addresses challenges in prostate SBRT planning, including metrics for target coverage, urethral sparing, OAR sparing, and normal tissue dose. Scorecards allow precise articulation of clinical intent, enabling quantification of trade-offs between competing objectives such as target coverage and OAR sparing. By assigning relative importance to different dosimetric goals, scorecards provide a comprehensive framework for evaluating plan quality and guiding optimization processes. Figure 1 illustrates the piecewise linear scoring functions used in the dosimetric scorecard.

### Model training and validation

Model training utilized 41 cases, with dosimetric scorecard guiding the optimization process. Scorecard was used to tune automatically generated optimization objective priorities for KBP model. This process involved creating initial plans, scoring them using scorecard, and then using these scored plans to train subsequent iterations of model. This iterative approach facilitates continuous improvement in plan quality as new models are trained from increasingly higher scoring plans to facilitate highly tuned objective priorities which enable single-click high-quality plan creation.

For validation, ten separate cases were created using KBP model with both VMAT and IMRT beam arrangements (five cases include rectum spacer). These cases were scored using dosimetric scorecard to assess model performance. This approach enables comprehensive evaluation of model’s ability to generalize to new cases while maintaining high plan quality with no user interaction during plan optimization.

### Model flexibility

**Fig. 5:**
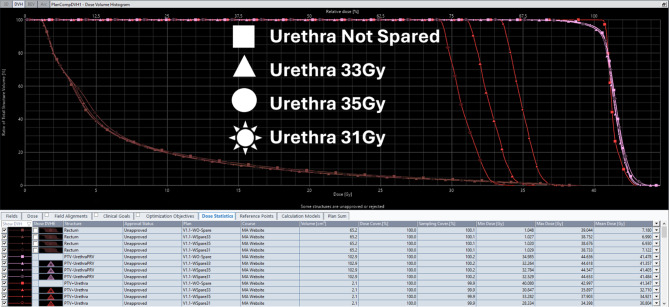
Example DVH with four plans at different levels of desired dose to the urethra: No sparing (40 Gy), 35 Gy, 33 Gy, and 31 Gy (non-SIB)

**Fig. 6:**
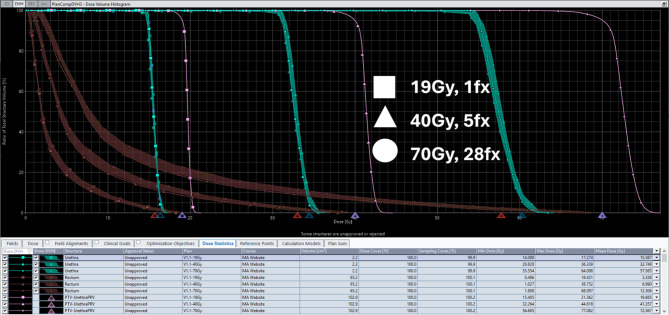
Multiple DVH and prediction bands plotted for different fractionation/prescription on a single representative case

ProstateSBRT-ARTIA-SIB40-36 Gy v1.1 model is intended to provide flexibility for various clinical scenarios. It supports two major targets: 40 Gy and 36.25 Gy treated as Simultaneous Integrated Boost. Model can be adapted for up to four dose levels: CTV-UrethraPRV = 40 Gy; PTV-UrethraPRV = 36.25 Gy; PTV+Rectum = 34.44 Gy (overlap region, if exists); PTV+Urethra = 33 Gy (default, other values accepted with doses scaled). Urethral dose can also be adjusted widely, from only managing hotspot overlap, to extreme urethral sparing by modifying the target dose for the ‘PTV+Urethra’ structure when applying the model. The model also supports no urethral structure sparing as described in it’s documentation. Clinical target volumes in the training set ranged from 17 to 117cc’s with an average of 63cc’s to ensure that there is flexibility to support various target/margin sizes. Figure 5 demonstrates the model’s flexibility in urethral dose sparing across different dose levels, and Fig. 6 shows the model’s adaptability to different fractionation schemes.

### Clinical validation from early adoption

Separately, the ProstateSBRT-ARTIA-SIB40-36 Gy v1.1 model was imported into an ARIA v18.0 clinical system for external validation at Kansas University Medical Center. A retrospective study was performed to test the functionality of the model and flexibility of urethra sparing; preference amongst treating physicians at the clinical site included ensuring dose coverage of the urethra to 36 Gy while restricting D0.03cc < 38 Gy for all prostate SBRT plans. The RapidPlan model objectives were adjusted to ensure more homogenous dose distributions to the targets than allowed by the ARTIA Trial, per treating physician request; upper limits were set to CTV-UrethraPRV of 104.5% with a priority of 350 to force dose homogeneity. Additional constraints were added to the UrethraPRV (max dose of 39.80 Gy), femoral heads (upper gEUD a = 40), and PTV+Urethra (upper constraint of 0.1% receiving 104%). Priority was increased to the line constraint (25 to 135) for the femoral heads to aid in achieving the clinical goal of V14.5 Gy < 5%. The rectum line constraint priority was decreased to 150.

Eight test patients were retrospectively replanned using the adjusted model on a Varian TrueBeam using three full arcs with collimators set to 15, 345, and 85 degrees. Comparisons with the original plans were performed using an adjusted dosimetric scorecard, which accounted for the institution’s preferences for PTV coverage and urethra sparing. This test validated both the model’s ability to adapt to different equipment (TrueBeam versus Halcyon) and different beam arrangements (three versus six arcs). The adjusted dosimetric scorecard allowed for a possible 182 points.

As an additional test, one patient was retrospectively planned with a simultaneous-integrated boost of 45 Gy to the GTV, with the CTV and PTV still receiving 40 Gy and 36.25 Gy, respectively. The goal of this exercise was to determine if an additional GTV boost could be integrated using the model. The CTV-UrethraPRV was cropped 2 mm away from the GTV to ensure homogeneous dose distribution within this region; since the GTV was over 2 mm away from the UrethraPRV structure, no additional cropping was performed to ensure the OAR tolerances were met. Additional scorecard objectives were added for the GTV, including V45 Gy and D99.9%, bringing the total possible points to 210.

### Statistical analysis

Statistical analysis was performed using Python with the SciPy and scikit-posthocs libraries. Paired t-tests and Wilcoxon signed-rank tests were used to compare paired data, with Cliff’s delta calculated to estimate the effect size. The Shapiro-Wilk test was used to test for normality of the differences in paired data. For comparisons of more than two groups, the Friedman test was used, followed by pairwise Wilcoxon tests with Holm-Bonferroni p-value correction for post-hoc analysis. A p-value of < 0.05 was considered statistically significant.

Treatment delivery times were estimated using the PlanDeliverySimulator ESAPI tool [17], which accounts for machine-specific parameters including gantry speeds, MLC constraints, and dose rates for both Halcyon and TrueBeam platforms.

## Results

### Model performance

ProstateSBRT-ARTIA-SIB40-36 Gy v1.1 demonstrated high-quality plan generation for validation set cases. Average score for validation cases showed significant improvement compared to previous average score of clinical cases (171.7/229 points) before they were manually improved and used as training set for KBP model. Table 1 shows the comparison between KBP-generated plans and original clinical plans for the validation cases.

**Table 1:** Comparison of dosimetric parameters between KBP-generated plans and original clinical plans (from ARTIA trial) for validation cases

Case #	Clinical Ethos (9 Field IMRT)	RapidPlan Halcyon (6 Arc VMAT)	RapidPlan Halcyon (19 Field IMRT)	RapidPlan TrueBeam M120 (3 Arc VMAT)	RapidPlan TrueBeam HDMLC (3 Arc VMAT)
23	157.64	201.45	183.39	193	199.64
24	161.57	188.56	180.7	187.8	195.29
25	184.76	212.08	191	208.47	208.58
26	175.47	177.42	172.28	186.54	188.11
27	178.86	204.18	196.23	197.15	203.13
Average	171.66	196.74	184.72	194.59	198.95

Scores out of 229 points. A Wilcoxon signed-rank test was used for p-value calculation. The p-values for comparing each RapidPlan configuration to Clinical Ethos are 0.062 (Halcyon VMAT), 0.125 (Halcyon IMRT), 0.062 (TrueBeam M120), and 0.062 (TrueBeam HDMLC)

### Model validation

Validation results showed excellent model generalizability across multiple delivery platforms and beam arrangements, as detailed in Table 2:

**Table 2:** Validation results for different delivery platforms halcyon 3 arc and 6 arc, halcyon/Ethos 19 fields, and TrueBeam/Edge 3 arcs for 10 validation cases

Case	Halcyon (3 Arc VMAT)			Halcyon (6 Arc VMAT)			Halcyon (19 Field IMRT)			TrueBeam M120 (3 Arc VMAT)			TrueBeam HDMLC (3 Arc VMAT)		
	MU	Time	Score	MU	Time	Score	MU	Time	Score	MU	Time	Score	MU	Time	Score
RQ00474-8	7043	540	197.44	6252	503	204.29	6838	703	187.19	5347	264	200.58	4791	258	205.91
RQ00475-3	5896	454	201.31	6098	507	203.86	6493	695	185.52	5054	256	199.90	5032	276	204.08
RQ00510-0	5644	435	189.30	5720	429	193.45	6824	722	173.68	4619	284	186.05	4588	263	193.09
RQ00514-4	5743	443	175.92	5790	459	176.76	7789	779	163.84	4847	251	174.55	4754	264	174.25
RQ00527-9	5923	456	200.77	5857	447	203.92	5917	648	190.08	4851	242	204.73	4518	256	202.18
23	4662	361	199.96	4878	393	201.45	6127	663	183.39	4551	230	193.00	4487	249	199.64
24	4694	364	188.57	4765	416	188.56	6311	689	180.70	4584	236	187.80	4757	261	195.29
25	5572	430	208.59	5572	447	212.08	6343	668	191.00	5487	265	208.47	5573	283	208.58
26	4777	371	167.55	4740	388	177.42	5980	644	172.28	5010	259	186.54	4828	284	188.11
27	5418	418	192.40	5211	423	204.18	5890	623	196.23	5167	284	196.23	4960	266	203.13
Average	5537	427	192.18	5488	441	196.59	6451	683	182.39	4952	257	193.79	4829	266	197.43

Time shown in seconds. Detailed dosimetric parameters are presented for clinically preferred configurations (6-arc and 19-field Halcyon, both TrueBeam variants) in Table 3

### Dosimetric comparison

**Fig. 2:**
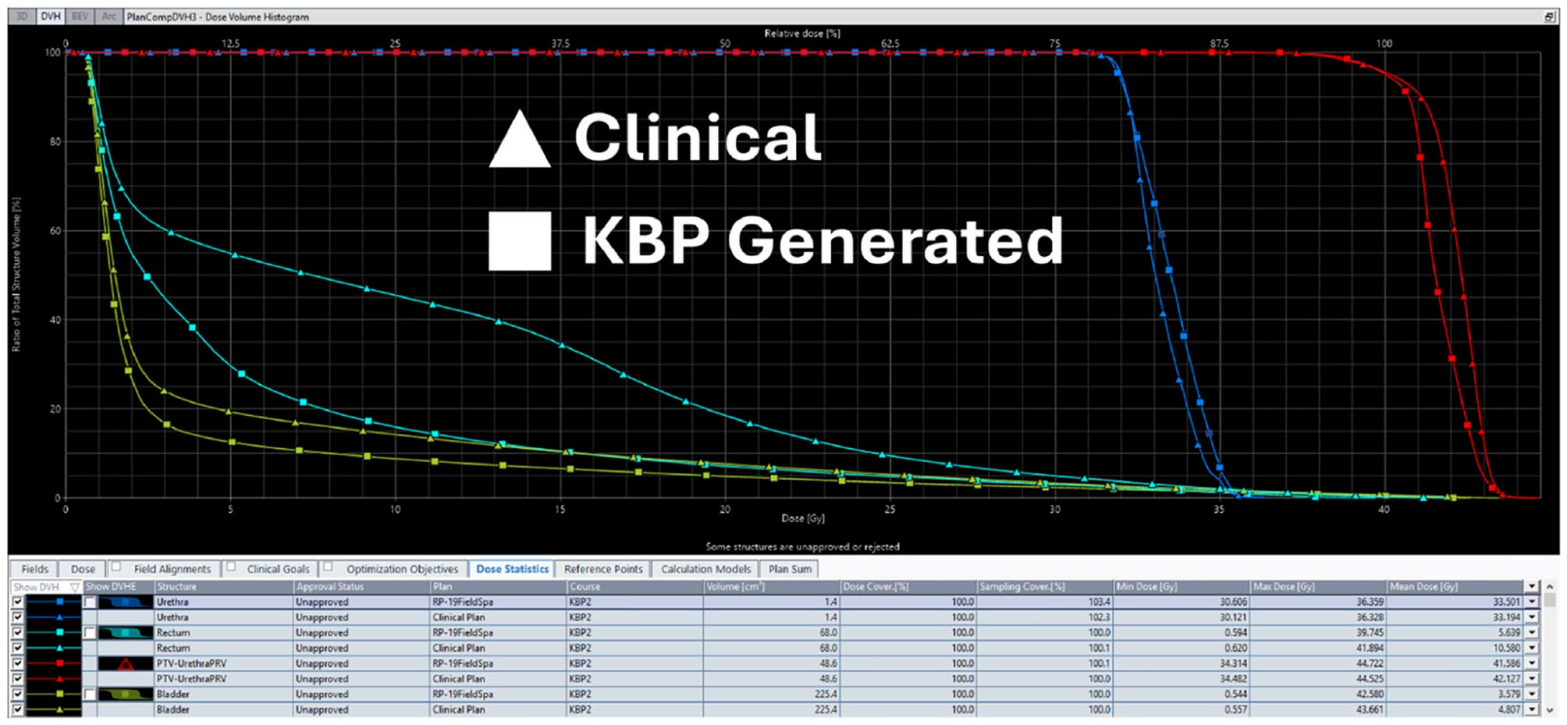
DVH comparison of KBP-generated plans (halcyon 6 arc) vs. original clinical plans for a representative validation case

**Fig. 3:**
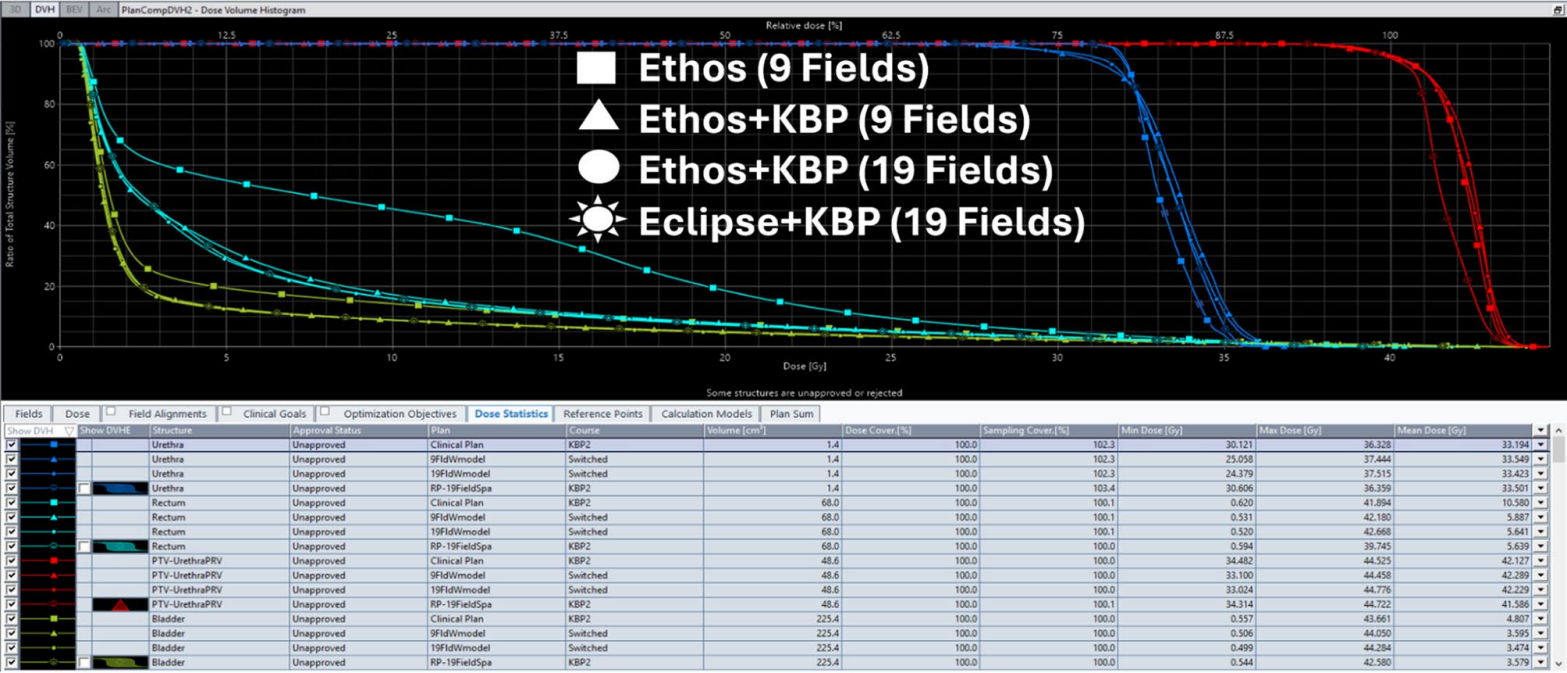
DVH for ethos 9 fields (178.86 points), Ethos+KBP 9 fields (182.49 points), Ethos+KBP 19 fields (186.90 points), and Eclipse+KBP 19 fields (199.41 points) plans for a validation case

Key dosimetric parameters were compared between KBP-generated plans and original clinical plans for both training and validation sets (Table 3). KBP model consistently produced plans with improved target coverage and OAR sparing. Figures 2 and 3 provide DVH comparisons illustrating the dosimetric improvements achieved with the KBP model.

**Table 3:** Key dosimetric parameters for different delivery platforms halcyon 6 arcs, halcyon/Ethos 19 fields, and TrueBeam/Edge 3 arcs for 10 validation cases

Platform/Parameter	RQ474-8	RQ475-3	RQ510-0	RQ514-4	RQ527-9	23	24	25	26	27	Average	Std Dev
**Halcyon (6 Arc VMAT)**												
PTV V36.25 Gy	96.15	95.69	95.46	96.55	96.46	96.90	96.53	97.06	97.11	95.20	**96.31**	**0.64**
Rectum V34.43 Gy	1.07	2.23	2.05	2.43	1.02	0.08	2.23	0.21	2.41	0.79	**1.45**	**0.86**
Bladder V32.62 Gy	4.35	8.55	8.86	18.81	7.87	10.88	8.31	1.83	5.74	3.33	**7.85**	**4.80**
**Halcyon (19 Field IMRT)**												
PTV V36.25 Gy	98.63	98.42	98.21	98.52	98.42	97.06	96.65	97.13	97.18	95.54	**97.58**	**1.42**
Rectum V34.43 Gy	1.27	1.26	2.41	2.51	1.15	0.07	1.86	0.18	2.29	1.24	**1.42**	**0.86**
Bladder V32.62 Gy	5.37	10.10	10.77	21.25	9.43	10.72	8.05	1.81	6.42	1.98	**8.59**	**5.57**
**TrueBeam M120 (3 Arc VMAT)**												
PTV V36.25 Gy	96.10	95.61	95.05	96.47	96.29	96.78	96.61	97.14	97.41	95.71	**96.32**	**0.77**
Rectum V34.43 Gy	1.05	0.97	1.97	2.48	0.96	0.10	1.89	0.24	3.33	1.75	**1.37**	**1.00**
Bladder V32.62 Gy	4.21	8.76	9.36	19.68	8.12	10.92	7.69	1.90	6.14	1.90	**7.87**	**5.12**
**TrueBeam HDMLC (3 Arc VMAT)**												
PTV V36.25 Gy	96.30	95.63	95.28	96.47	96.31	96.69	96.25	96.97	96.96	94.95	**96.18**	**0.82**
Rectum V34.43 Gy	1.07	2.61	2.12	2.61	0.95	0.09	1.87	0.23	2.24	0.82	**1.46**	**0.92**
Bladder V32.62 Gy	4.36	8.69	9.33	19.07	8.01	10.89	7.66	1.76	6.08	3.26	**7.91**	**4.88**

A Friedman test showed a statistically significant difference across platforms for all three parameters (p < 0.001). Halcyon 3-arc parameters omitted as they show similar treatment times to 6-arc configurations

### Separate clinical data analysis and impact from early adopter site

Initial clinical testing shows plans were able to ensure excellent coverage of the PTV and CTV-UrethraPTV, yielding V36.25 Gy and V40 Gy of 99.1 ± 0.5% and 93.2 ± 2.1%, respectively. In accordance with planning preferences, urethra and urethraPRV structures were kept to 37.8 ± 0.2 Gy and 39.9 ± 0.2 Gy, respectively, across the clinical test patient cohort. The model-generated plans outperformed manually optimized plans, scoring 161.4 ± 6.2 (88.7%) versus 144.2 ± 8.3 (79.2%) out of a possible 182 points on the adapted clinical scorecard, representing a 9.5% improvement in overall plan quality.

As compared to the manually optimized clinical plans, plans using the model scored higher in each case, averaging 161.4 ± 6.2 (88.7%) out of a possible 182; manually optimized plans averaged 144.2 ± 8.3 (79.2%), a 9% lower score. In comparing KBP versus manually optimized plans, key improvements were seen in CTV-UrethraPRV (3% increase in V40 Gy), rectum (31.2% reduction in V10 Gy), bladder (2.9cc improvement in V32.62 Gy), and PTV_Ring (1.32 Gy reduction in D0.03cc), as detailed in Tables 4 and 5.

**Table 4:** Combined validation results: clinical comparison with complexity metrics for all delivery platforms

Case	Clinical Ethos Score	Halcyon (3 Arc)			Halcyon (6 Arc)			Halcyon (19 Field)			TrueBeam M120			TrueBeam HDMLC		
		MU	Time	Score	MU	Time	Score	MU	Time	Score	MU	Time	Score	MU	Time	Score
RQ00474-8	-	7043	540	197.44	6252	503	204.29	6838	703	187.19	5347	264	200.58	4791	258	205.91
RQ00475-3	-	5896	454	201.31	6098	507	203.86	6493	695	185.52	5054	256	199.90	5032	276	204.08
RQ00510-0	-	5644	435	189.30	5720	429	193.45	6824	722	173.68	4619	284	186.05	4588	263	193.09
RQ00514-4	-	5743	443	175.92	5790	459	176.76	7789	779	163.84	4847	251	174.55	4754	264	174.25
RQ00527-9	-	5923	456	200.77	5857	447	203.92	5917	648	190.08	4851	242	204.73	4518	256	202.18
23	157.64	4662	361	199.96	4878	393	201.45	6127	663	183.39	4551	230	193.00	4487	249	199.64
24	161.57	4694	364	188.57	4765	416	188.56	6311	689	180.70	4584	236	187.80	4757	261	195.29
25	184.76	5572	430	208.59	5572	447	212.08	6343	668	191.00	5487	265	208.47	5573	283	208.58
26	175.47	4777	371	167.55	4740	388	177.42	5980	644	172.28	5010	259	186.54	4828	284	188.11
27	178.86	5418	418	192.40	5211	423	204.18	5890	623	196.23	5167	284	196.23	4960	266	203.13
Avg	171.66^*^	5537	427	192.18	5488	441	196.59	**6451**	**683**	**182.39**	**4952**	**257**	**193.79**	**4829**	**266**	**197.43**

Time shown in seconds. MU = Monitor Units. Scores out of 229 points

^*^ Clinical Ethos average for cases 23-27 only (n=5)

Demonstrates complexity comparison: TrueBeam platforms achieve similar scores with lower MU and faster delivery times compared to Halcyon configurations

**Table 5:** Clinical site key dosimetric comparisons - model versus manual plans

StructureId	Metric Text	Model Avg	Manual Avg	p-value	Cliff’s Delta
PTVpsv	Volume at 36.25 Gy [%]	99.09	98.54	0.039	0.625 (large)
PTV+Urethra	Dose at 99.9% [Gy]	36.05	35.67	0.055	0.656 (large)
CTV-UrethraPRV	Volume at 40 Gy [%]	93.56	90.60	0.148	0.312 (small)
CTV-UrethraPRV	Dose at 99.9% [Gy]	38.89	38.44	0.039	0.469 (medium)
Rectum	MeanDose [Gy]	6.55	11.13	0.008	−1.000 (large)
Rectum	Volume at 36.25 Gy [cc]	0.45	0.56	0.641	−0.031 (negligible)
Rectum	Volume at 34.43 Gy [cc]	0.88	1.29	0.008	−0.469 (medium)
Rectum	Volume at 10 Gy [%]	18.36	49.59	0.008	−1.000 (large)
Rectum	Dose at 50% [Gy]	4.18	9.51	0.008	−0.969 (large)
Rectum	Dose at 20% [Gy]	9.33	18.77	0.008	−1.000 (large)
Bladder	MeanDose [Gy]	6.38	7.85	0.008	−0.188 (small)
Bladder	Volume at 32.62 Gy [cc]	9.60	12.50	0.016	−0.500 (large)
Bladder	Volume at 15 Gy [%]	14.71	20.24	0.008	−0.219 (small)
Bladder	Dose at 40% [Gy]	3.69	5.45	0.008	−0.219 (small)
Urethra	Dose at 0.03cc [Gy]	37.83	38.14	0.383	−0.219 (small)
PTV_Ring	Dose at 0.03cc [Gy]	33.76	35.08	0.039	−0.500 (large)

p-values calculated using Wilcoxon signed-rank test

**Fig. 4:**
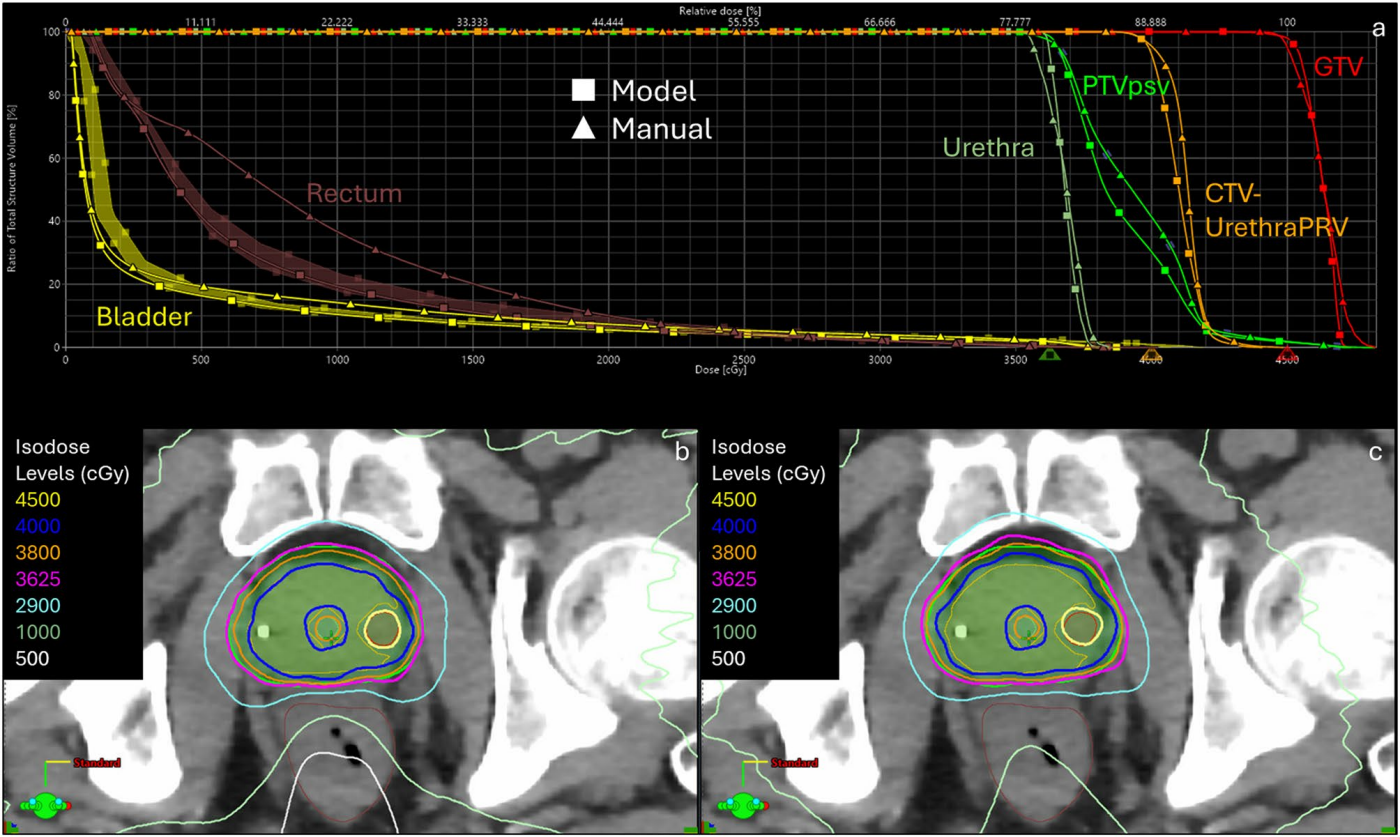
DVH comparison (**a**) between model-based and manual optimization for a prostate SBRT case involving a 45 Gy GTV sib. The model plan (**b**) shows improved coverage of the prostate PTV and GTV and improved sparing of OARs, including the rectum, as compared to the manually optimized plan (**c**)

For the case involving a GTV boost to 45 Gy, the model-based plan outscored the manual plan by nearly 8 points (186.3 (88.7%) to 178.4 (85%), respectively) due to improved coverage and sparing of the rectum and bladder, especially at low and intermediate dose levels. The model was able to accurately predict OAR dose; the rectum and bladder were able to align to the lower border of the DVH prediction despite introducing the GTV boost of 45 Gy into the optimizer. Figure 4 shows the DVH comparison for this GTV boost case.

As for the clinical cases, four patients were planned with the standard 40/36 Gy dose schema. The fifth case treated the PTV to 36.25 Gy and boosted a GTV to 42.5 Gy (no CTV-UrethraPRV dose level). PTV V36.25 Gy averaged 98.3 ± 1.2% across the clinical cases, with CTV-UrethraPRV netting an average V40 Gy of 97 ± 2%; for the case with the GTV SIB boost, a D99% of 96% was achieved, while the urethra was spared to a D0.03cc of 37.7 Gy. For the case involving a GTV boost, the GTV was matched to the CTV-UrethraPRV structure during plan optimization. Plan scores closely matched the test plans, yielding an average score of 154.9 ± 7.8 (85.1%) as shown in Table 6.

**Table 6:** Clinical site - scores by patient (out of 182)

Case	Model Score		Manual Score		Δ **Score**
	Total	%	Total	%	(%)
Test1	158.32	87.0%	145.06	79.7%	7.3%
Test2	148.56	81.6%	130.80	71.9%	9.8%
Test3	168.49	92.6%	162.16	89.1%	3.5%
Test4	160.74	88.3%	146.98	80.8%	7.6%
Test5	165.63	91.0%	145.42	79.9%	11.1%
Test6	167.25	91.9%	151.09	83.0%	8.9%
Test7	164.42	90.3%	142.87	78.5%	11.8%
Test8	157.66	86.6%	139.16	76.5%	10.1%
GTV SIB (out of 210)$^*$	186.30	88.7%	178.40	85.0%	3.7%
Average (Std Dev)	161.38 (6.13)	88.7%	145.44 (8.47)	79.9%	8.8%

^*^ The GTV SIB case was excluded from the statistical analysis

A Wilcoxon signed-rank test showed that the model scores were significantly higher than the manual scores (p = 0.008)

The adjusted ProstateSBRT-ARTIA-SIB40-36 Gy v1.1 model was made clinically available for planners to employ on SBRT prostate cases after validation testing at the early adopter’s site. Initial cases were planned using varied prescription schemes: 40/36 Gy (four cases) and 42.5(GTV SIB)/36 Gy (one case out of 210 points for boost volume). Dosimetric scorecard analysis was run to assess the quality of all five plans.

## Discussion

While KBP models have improved efficiency in radiotherapy planning, creating a model that effectively balances urethral sparing with target coverage in prostate SBRT has remained challenging. Most existing models lack the adaptability required for different dose prescriptions and clinical scenarios. The ProstateSBRT-ARTIA-SIB40-36 Gy v1.1 model addresses these limitations. Utilizing a high-quality iteratively improved knowledge base for training allows this model to create consistent, high quality plans, without requiring user interaction across diverse treatment settings which is useful both in the clinic and for automated plan generation.

### Creating a flexible, shared, single click RapidPlan model

Developing this prostate SBRT model with urethral sparing demonstrates an effective method for creating highly flexible KBP models. Scorecard guided iterative improvement of plans used for model training, resulted in a KBP model that effectively captured the dosimetric preferences between geometric features and dose objectives for urethral, rectum and bladder sparing. This approach enabled the development of a highly specialized model which is also capable of adapting to different dose prescriptions, including little or no urethral sparing while maintaining consistent plan quality across diverse geometric scenarios and target volumes. The resulting model qualitatively reduces planning time while maintaining or improving plan quality, with demonstrated adaptability to different delivery techniques and dose prescriptions.

The development of such adaptable models is particularly valuable for prostate SBRT, where protocols and dose prescriptions continue to evolve, and the precise balance between target coverage and urethral sparing remains a consideration.

Both our model and scorecard are publicly available, and are able to perform urethral sparing or not as indicated. Other models such Scaggione et al. 2024 and 2018 provided excellent evidence of time savings and adapting prior models to prostate SBRT. [3, 5, 6, 18] However, these prior models were not made publicly available, didn’t demonstrate single click non-interactive use or demonstrate such flexibility in prescription or structure requirements such as optional urethral sparing.

### Clinical impact and future directions

Developing high-quality KBP models guided by comprehensive dosimetric scorecards has potential to significantly impact clinical practice. The early qualitative results from a single site clinical implementation demonstrate the potential for reduction in the number of planning cycles.

Flexibility built into models like ProstateSBRT-ARTIA-SIB40-36 Gy v1.1 allows easy adaptation to different clinical scenarios or evolving treatment paradigms. This model’s adaptability increases likelihood of longer term clinical relevance as practices change over time. Systematic reviews have also shown that restricting urethral Dmax to ≤ 90 Gy EQD2 significantly lowers genitourinary toxicity without compromising oncologic control [19]. Historically, planners would consider that increased urethral sparing would result in greater dose heterogeneity within the target volume which often required additional tuning of optimization objectives before the availability of freely shared, highly tuned KBP solutions.

Future work should focus on further quantifying clinical benefit. The iterative nature of model development, guided by scorecard evaluation, can lead to overall improvements in plan quality compared to manual planning [2]. The presented model demonstrates the power of training from high quality plans. Additionally, the incorporation of boosts to gross disease volumes, as demonstrated by our model in a single case, warrants further study and characterization [20].

Similar highly tuned, single-click based KBP models have been used in large scale synthetic data generation workflows used to bulk create high quality dose data for training neural networks [14].

Exploring use of dosimetric scorecards as cohort-specific high-level utility functions for direct optimization in DVH space represents an avenue for future research and could lead to even more efficient planning processes.

### Limitations

There are limitations to this current study. The current study had a small validation set of only 10 patients (half with a rectal spacer). This model was only validated at one external institution. The iterative training process naturally creates a bias toward plans that optimize scorecard performance, making statistical significance testing of scorecard improvements potentially circular. However, the statistical analysis confirms significant improvements in key clinical metrics including rectum sparing (V10Gy reduction, *p* = 0.008) and bladder sparing (V32.62 Gy improvement, *p* = 0.016).

## Conclusion

ProstateSBRT-ARTIA-SIB40-36GyV1.1 KBP model, developed using dosimetric scorecard-guided approach, demonstrates the ability to generate high-quality prostate SBRT plans with optional urethral dose sparing. We have demonstrated flexible performance and ease of clinical implementations as demonstrated in our validation results using a variety of treatment techniques. Initial evaluation and clinical implementation has been successful at the University of Kansas Medical Center. The development and validation of such models represent significant steps towards more consistent, high-quality, and efficient radiotherapy treatment planning, with potential to improve outcomes for patients undergoing prostate SBRT and other complex treatments.

## Data Availability

Links to the developed rapid plan model are available within the manuscript https://medicalaffairs.varian.com/pelvis-prostateSBRT-vmat2.

## Code availability

The dosimetric scorecard implementation and analysis scripts are available on Varian Medical Affairs website [15, 16].

## Material availability

RapidPlan model available for public download https://medicalaffairs.varian.com/pelvis-prostateSBRT-vmat2.

## Declarations

### Supplementary information

Supplementary materials such as the RapidPlan model, example cases, and full dosimetric scorecard are available on the Varian Medical Affairs website https://medicalaffairs.varian.com/pelvis-prostateSBRT-vmat2.
